# Iron Oxide Mediated Photothermal Therapy in the Second Biological Window: A Comparative Study between Magnetite/Maghemite Nanospheres and Nanoflowers

**DOI:** 10.3390/nano10081548

**Published:** 2020-08-07

**Authors:** Sonia Cabana, Alberto Curcio, Aude Michel, Claire Wilhelm, Ali Abou-Hassan

**Affiliations:** 1 Laboratoire de PHysico-chimie des Électrolytes et Nanosystèmes InterfaciauX (PHENIX), CNRS UMR8234, Sorbonne Université, F-75252 Paris CEDEX 05, France; scabmon@gmail.com (S.C.); aude.michel@sorbonne-universite.fr (A.M.); 2 Laboratoire Matière et Systèmes Complexes, CNRS UMR 7057, Université de Paris, 10 rue Alice Domon et Léonie Duquet, 75205 Paris CEDEX 13, France; alberto.curcio@univ-paris-diderot.fr

**Keywords:** magnetic nanoparticles, second biological window, photothermia, nanothermal therapies, magnetic nanoflowers

## Abstract

The photothermal use of iron oxide magnetic nanoparticles (NPs) is becoming more and more popular and documented. Herein, we compared the photothermal (PT) therapy potential versus magnetic hyperthermia (MHT) modality of magnetic nanospheres, largely used in the biomedical field and magnetic multicore nanoflowers known among the best nanoheaters. The NPs were imaged using transmission electron microscopy and their optical properties characterized by UV-Vis-NIR-I-II before oxidation (magnetite) and after oxidation to maghemite. The efficiency of all NPs in MHT and PT in the preferred second near-infrared (NIR-II) biological window was carried out in water and in cancer cells. We show that, in water, magnetite nanoflowers are the most efficient nanoheaters for both modalities. Moreover, PT appears much more efficient than MHT at low NP dose, whatever the NP. In the cellular environment, for PT, efficiency was totally conserved, with magnetite nanoflowers as the best performers compared to MHT, which was totally lost. Finally, cell uptake was significantly increased for the nanoflowers compared to the nanospheres. Finally, the antitumor therapy was investigated for all NPs at the same dose delivered to the cancer cells and at reasonable laser power density (0.3 W/cm^2^), which showed almost total cell death for magnetite nanoflowers.

## 1. Introduction

Iron oxide magnetic nanoparticles (MNPs), such as magnetite (Fe_3_O_4_) and its oxidized form maghemite (γ-Fe_2_O_3_), emerged as promising nanotheranostic agents due to their versatile magnetic properties, their biocompatibility, and their biodegradability [1]. Consequently, MNPs have made their way into different applications in the biomedical field including, among others, MRI contrast agents [2], drug delivery [3], tissue engineering [4,5], magnetic targeting [6,7,8,9], and as heat mediators in magnetic hyperthermia (MHT) cancer therapy [10,11]. Unlike other thermal nanotherapies, MHT can be used non-invasively at any depth in tissues, but it still suffers from major restrictions mainly due to the low yield of heat generated per mg. Consequently, several approaches have been suggested to overcome these limitations: among them, one is based on the synthesis of novel nanostructures having an optimized heating [12,13,14]; another consists of the association of MNPs with other heat-generating materials, such as plasmonic ones, specifically designed for photothermal (PT) therapy, resulting in a multifunctional magneto-plasmonic nanohybrid platform. Such plasmonic materials include metals, such as gold (Au), providing the hybrids with an absorption in the first near-infrared (NIR-I) optical window in biological tissues or semiconductors, such as copper sulfide (CuS), which possesses a strong absorption in the second (NIR-II) window [15,16,17,18]. Despite the versatility of these approaches, synthesis and optimization of any new structure is both time- and money-consuming, can be difficult to control, and the biocompatibility of the final structures is not always very studied, which may limit the applications.

Another approach which could also overcome these limitations has more recently been reported, based on exploiting the intrinsic optical properties of the MNPs resulting from the electronic transitions in magnetic iron oxides. These transitions elicit a spectral absorption localized in the near-infrared (NIR) therapeutic window and thus they are useful for applications in PT, endowing the MNPs with a second heating modality in addition to MHT [19,20,21,22]. Consequently, magnetic nanoparticles that display the dual functions of magnetism and NIR absorption are now being pursued. For instance, synthetic magnetic nanostructures [23] or biogenic magnetosomes [24] have been used in the NIR-I region as MRI/photothermal theranostic agents as well as for multivalent thermal cancer treatment, respectively. In particular, the magnetosomes were tagged with RGD peptide by genetic engineering, so that, combined with a targeting potential, they could generate photothermal heat in vivo at therapeutic levels after systemic administration, successfully inhibiting tumor progression. The other still rare examples showing magnetic nanoparticles for PT applications are systematically based on their photothermal response to an excitation in the NIR-I range [20,23,25]. However, the second region (NIR-II) is preferred for biomedical applications since it offers enhanced tissue penetration due to reduced scattering losses and consequently higher heating efficiency [21]. The first example on using an iron band in the NIR-II for PT applications has been reported by Huang et al. [26]. Magnetite aggregates/clusters, prepared using a hydrothermal method, displayed an absorption band in the NIR-II and in turn showed significant photothermal conversion upon irradiation by a 1064 nm laser, and the ability to magnetically assist the photothermal ablation of cancer cells [26]. In another, later example, the green synthesis of 15 nm eugenate-capped iron oxide nanoparticles described by Kharey et al. [27] achieved important heat generation upon laser irradiation at 1060 nm wavelength. To the best of our knowledge, these reports are so far the only two that show the potential of magnetite nanoparticles as photothermal agents in the second NIR window. The shape of the magnetic core, its crystallinity as well as the crystal phase (magnetite versus maghemite for instance), have not yet been quantified, neither intrinsically for the photothermal modality, nor comparatively with most of the used magnetic hyperthermia modalities.

In this study, we provide a detailed comparative analysis of the thermal potential in both MHT and PT modalities of two differently shaped MNPs, rock-like nanospheres, and multicore magnetic nanoflowers. The rock-like nanospheres obtained by co-precipitation are among the most studied NPs in the field of biomedical applications with high biocompatibility, as, for instance, tested within human mesenchymal stem cells [4,28], while the nanoflowers obtained by the polyols process are among the best known nanoheaters in MHT [29,30,31]. The optical properties of the as-prepared magnetite NPs showed the presence of an intervalence charge transfer band (IVCT) in the NIR-II window, which disappeared in the oxidized maghemite form. Consequently, the thermal potential of all nanoparticles was evaluated by MHT and PT in the NIR-II, both in solution and after cancer cell incorporation. Taken together, the results demonstrate clearly the predominance of magnetite performance with respect to maghemite for both therapies, but especially for the photothermal one, as well as the predominance of the multi-core structure for both therapies, but especially for the magnetic hyperthermia one. It also evidences the relevance of applying PT only in the cellular environment, where magnetic hyperthermia is totally inhibited. Finally, it demonstrates the enhanced cellular uptake for the magnetic nanoflowers. As a result, for the same dose administered, the magnetite nanoflowers only could achieve a complete cancer cell destruction, and this, quite remarkably, in the NIR-II window, and at a laser power density of 0.3 W/cm^2^, for which no side effects were recorded on control cells.

## 2. Materials and Methods

### 2.1. Materials

All reagents were of analytical purity and used without further purification. Nitric acid (HNO_3_, 70%) was obtained from VWR (Paris, France). Ethyl acetate (>99.5%), acetone (technical grade), ethanol (96%), diethyl ether (100%), N-methyldiethanolamine (NMDEA, 99%), and diethylene glycol (DEG, 99%) were obtained from Sigma-Aldrich (Saint Quentin Fallavier, France). Sodium hydroxide (NaOH, 98%), iron(III) nitrate nonahydrate (Fe(NO_3_)_3_•9H_2_O, >98%), and iron(II) chloride tetrahydrate (FeCl_2_•4H_2_O, 98%) were from Alfa Aesar. Iron(III) chloride hexahydrate (FeCl_3_•6H_2_O, >97%) was obtained from Panreac (Lyon, France).

### 2.2. Synthesis of Iron Oxide Nanoparticles

**Nanoflowers:** magnetite Fe_3_O_4_ nanoflowers (IONFs) were prepared via a polyol process [32]. In brief, 80 mL of a 1:1 *v/v* mixture of DEG and NMDEA was flushed with nitrogen in a three-neck round-bottom flask while magnetically stirred for 1 h under inert atmosphere. Then, 1.08 g (4 mmol) of FeCl_3_•6H_2_O and 0.40 g (2 mmol) of FeCl_2_•4H_2_O were allowed to solubilize overnight. Meanwhile, 0.64 g (16 mmol) of NaOH was dissolved under magnetic stirring in 40 mL of a mixture of DEG and NMDEA (1:1 *v/v* volume ratio) in a separate flask. The NaOH solution was flushed with nitrogen for 1 h before being combined with the iron(II)/iron(III) mixture. The color immediately turned from yellow to dark green. The flask containing the resulting solution was then placed in an oil bath and heated up to 220 °C by an electronically controlled heating plate (model C-MAG HS 7, IKA) before allowing the reaction to occur for an hour under magnetic stirring and then left to cool down at room temperature. The formed magnetic nanoparticles were then collected using a strong permanent magnet and washed several times with a mixture of ethanol and ethyl acetate (1:1 *v/v*), once with 10% nitric acid, twice with acetone, and twice with diethyl ether in order to eliminate organic and inorganic impurities. IONFs were then readily redispersed in water by stirring in open air to remove volatile solvents. At this stage, a black monophasic dispersion of IONFs was obtained. In order to obtain maghemite (γ-Fe_2_O_3_) nanoflowers, 8.6 g of iron(III) nitrate was added to the monophasic dispersion of IONFs as a strong oxidant and heated at 80 °C for 45 min while mechanically shaking [33]. The colloidal solution then turned from clear black to mat brown-orange. The oxidized IONFs were flocculated by the addition of 10% nitric acid, washed twice with acetone and twice again with diethyl ether, and finally resuspended in water. At this stage, a deep orange-black dispersion of maghemite IONFs was obtained.

**Nanospheres:** magnetite Fe_3_O_4_ nanospheres were synthesized by alkaline coprecipitation of Fe^3+^ and Fe^2+^ precursors following Massart’s procedure [34]. Briefly, 0.9 mol of FeCl_2_•4H_2_O and 1.6 mol of FeCl_3_•6H_2_O were mixed with 7 mol of alkaline solution (NH_4_OH). At the end of the reaction, the obtained magnetic nanoparticles were separated from the supernatant by magnetic collection and washed several times with nitric acid, acetone, and diethyl ether to remove unreacted molecules and by-products. Maghemite (γ-Fe_2_O_3_) nanospheres were obtained by mixing the magnetite nanoparticles with a boiling solution of 0.8 mol of ferric nitrate (Fe(NO_3_)_3_) for 30 min. Then, the nanoparticles were magnetically collected and 2 L of distilled water and 360 mL of 20% nitric acid were added and the mixture was stirred for 10 min. After several washing steps in acetone and diethyl ether to remove the excess ions, the maghemite nanospheres were resuspended in water.

At the end of each preparation, nanoparticles were functionalized with citrate by mixing with sodium citrate at a molar ratio of 0.13 (mol Fe/mol citrate) and heated up to 80 °C for 30 min to promote absorption of citrate anions onto their surface [34].

### 2.3. Elemental Analysis

The concentration of Fe^2+^ was determined using the phenanthroline method as described by Gorski et al. [35] after dissolving pre-weighted samples in 5 M degassed HCl in the anaerobic glovebox. The total iron concentration of nanoparticle suspensions or nanoparticle-containing cells was analyzed by inductively coupled plasma atomic emission spectrometry (ICP-AES, Agilent, Santa Clara, CA, USA). The samples were digested in pure 69% HNO_3_ overnight and then diluted up to 2% HNO_3_ in ultrapure H_2_O prior to the analysis.

### 2.4. UV-Vis-NIR-I-II Spectroscopy

Optical absorption spectra were recorded using AvaSpec-ULS 2048L (Avantes, Vanves, France) in the 200–1300 nm wavelength range.

### 2.5. Magnetic Hyperthermia and Photothermia Measurements

All measurements of nanoparticles in aqueous dispersion and within cells were performed in 0.5 mL tubes containing a volume of 50 µL and placed in a thermostat device connected to a water bath at adjustable temperature. For magnetic hyperthermia, an nB Nanoscale Biomagnetic device was used to generate a 180-Gauss alternating magnetic field at a frequency of 470 kHz. For photothermia, a 1064 nm laser (Laser Components S.A.S, France) was used to irradiate the sample from above at a distance of 4.5 cm from the center of the suspension and at power density of 0.3 W cm^−2^. The heating process was carried out for 10 min. For both the heating methods, the sample temperature was recorded with an infrared thermal camera (FLIR SC7000, Croissy-Beaubourg, France) operating at 1 frame per second, controlled with the software ALTAIR (version 4.1.0, Pairs, France).

The specific loss power (SLP), specified as the power dissipation per unit mass of iron (W g^−1^), and often also denoted as the specific absorption rate (SAR), was calculated using the following formula:

(1) $$SLP=\frac{C\;V}{m}\;\frac{dT}{dt}$$

where *m* is the total mass of iron in the sample, *C* is the specific heat capacity of the sample (C_water_ = 4185 J L^−1^ K^−1^), *V* is the sample volume (50 μL), and d*T*/d*t* is the temperature increase at the initial linear slope (over the first 30 s).

### 2.6. Cell Culture and Internalization Assays

For the in vitro measurements, PC3 human prostate cancer cells were cultured in Dulbecco’s modified Eagle’s medium (DMEM) supplemented with 10% fetal bovine serum (FBS) (Sigma Aldrich, Saint Quentin Fallavier, France) and 1% penicillin, and maintained at 37 °C with 5% pCO_2_ and 95% relative humidity. At 90% of confluence, the cells were incubated for 30 min with the 4 magnetic nanoparticle preparations (magnetite nanospheres, maghemite nanospheres, magnetite nanoflowers, and maghemite nanoflowers) dispersed in serum-free RPMI-1640 medium (Sigma Aldrich, Saint Quentin Fallavier, France) supplemented with 5 mM sodium citrate and previously sonicated for 5 min (45 kHz). The iron concentration in the medium ranged from 10 to 2500 µM. At the end of the incubation, the medium was removed and the cells were rinsed with culture medium, and further incubated with fresh medium at 37 °C for an additional 2 h to remove any non-internalized nanomaterial. Therefore, the cells were detached by trypsinization, counted, and further analyzed. 

### 2.7. Cytotoxicity Study

Photothermia treatment was carried out on the magnetic nanoparticle-containing PC3 cancer cells (TCC^®^CRL-1435™) at 37 °C. Right after the treatment, the cells were seeded back in 24-well plates and the cytotoxicity study was performed 24 h later. The first analysis consisted of the evaluation of the treatment impact on the mitochondrial metabolic activity using resazurin-based assay (Alamar Blue, Invitrogen, France). The treated cells and the corresponding non-irradiated cells in each condition were incubated with colorless DMEM, without phenol red supplemented with 10% Alamar Blue. After 1 h at 37 °C, 100 μL of the solution was transferred to each well of a 96-well plate and the florescence at 585 nm was measured by a plate reader (Enspire, Perkin Elmer) and normalized against the non-treated control. The second method assessed the proportion of necrotic/apoptotic cells using an apoptosis detection kit (APC-annexin V with propidium iodide, Biolegend) by flow cytometry. Annexin V and propidium iodide were added to 100 μL of cell suspension containing 5 × 10^5^ cells following manufacturer’s instructions. After 15 min of incubation, annexin V binding buffer was added to each tube. Stained cells were analyzed with a CyAn ADP 9C flow cytometer (Beckman Coulter).

## 3. Results

**Figure 1:**
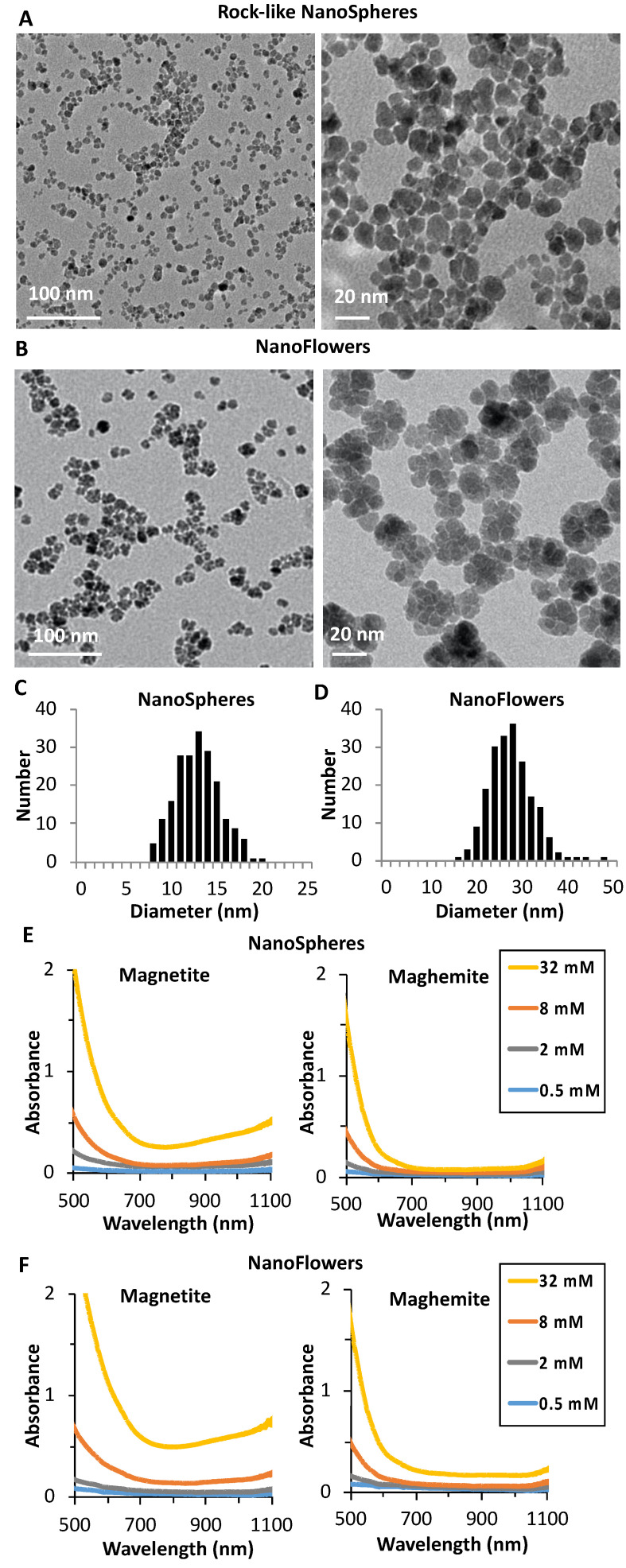
Low and high magnification TEM images of (**A**) the rock-like shape magnetic nanoparticles (MNPs) (nanospheres) and (**B**) the multicore MNPs (nanoflowers); size distribution histogram of (**C**) the nanospheres and (**D**) the nanoflowers; UV-Vis-NIR-I-II spectrum upon dilution from 32 to 0.5 mM before (magnetite) and after oxidation (maghemite) of the (**E**) nanospheres and (**F**) nanoflowers.

Figure 1A,B present low and high magnification TEM images of the synthesized nanoparticles, nanospheres, and nanoflowers, respectively. The rock-like shaped MNPs synthesized using the well-established Massart process are single-core and mostly spherical (nanospheres) with an average diameter of 12.7 ± 2 nm (Figure 1C). The nanoparticles obtained using the polyols process are flower-shaped (nanoflowers) multicores with an average size of 27 ± 4 nm as deduced from TEM images (Figure 1D). For all nanoparticles, their average size and shape did not show any modifications by TEM analysis before and after oxidation. The crystallinity of all NPs was checked using powder X-ray diffraction, which showed for all nanoparticles the typical Bragg peaks of the oxide spinel structure similar to magnetite and maghemite. The size distribution by TEM and from the line width of the (311) plane reflection obtained by XRD using Scherrer equation were compared. For the nanoflowers, the XRD gives the size of the small grains (cores) composing the nanoflowers, which was almost identical to what was observed by TEM (about 11 nm); however, in the case of the spherical nanoparticles, the diameter as deduced from XRD (10.6 nm) was below the physical size obtained by TEM, which shows that magnetic nanospheres are less crystalline than the magnetic nanoflowers. It is worth mentioning that, for the nanoflowers, although they are multicores, they are single-crystalline and constituted of small grains with the same crystalline orientation as was demonstrated elsewhere [29,30]. To determine the chemical composition of all nanoparticles, freshly prepared or after forced oxidation, we used a colorimetric approach based on the phenanthroline method to determine the concentration of Fe^2+^ [35]. The total concentration of iron was determined by atomic absorption. Before oxidation, the ratio of Fe^2+^ to Fe^3+^ (x) was very close to the predicted stoichiometry (x = 0.50) of magnetite in both types of nanoparticles (x = 0.47 and 0.49 for nanospheres and nanoflowers, respectively). After forced oxidation, the ratio decreased to 1/60 in both structures, which shows that oxidation was essentially complete and the analyzed products can be regarded as maghemite. The optical properties of all synthesized nanoparticles, freshly prepared, were analyzed by UV-Vis-NIR-I and II spectroscopy before and after forced oxidation (Figure 1E,F). All non-oxidized nanoparticles (Figure 1E,F(left)) featured a “U-shape” absorption spectrum, which persisted after dilution from 32 to 0.5 mM. Additionally, an absorption band at over 1000 nm could be observed (Figure 1E,F(right)), which disappeared after oxidation, indicating that non-oxidized MNPs can absorb NIR-II photons. This band can be attributed to the intervalence charge transfer, which is a fingerprint of stoichiometric or near-stoichiometric magnetite phases [36]. The origin of this absorption in magnetite is due to its crystal structure. Indeed, Fe_3_O_4_ is a conductive mixed-valence iron oxide that has an inverse spinel structure with Fe(II) and Fe(III) in the octahedral sites of the crystal lattice unit cell [37]. This distribution of mixed oxidation states produces a d-d type charge transfer, which in turn gives rise to an absorption in the NIR-II region at 1000–1350 nm [36,38]. After the forced oxidation, the oxygen deficiency and the abundance of Fe(III) ions at the octahedral sites in the spinel structure caused the loss of the intervalence transition, resulting in the color shift of the suspension to red-orange [39,40]. Both nanospherical and nanoflower non-oxidized suspensions maintained their “U-shaped” absorption curve over 2 months of storage at 4 °C, which was encouraging to test further their potential as photothermal agents. For more simplicity, in the rest of the manuscript, we will refer to the non-oxidized phase as magnetite and to the oxidized one as maghemite.

**Figure 2:**
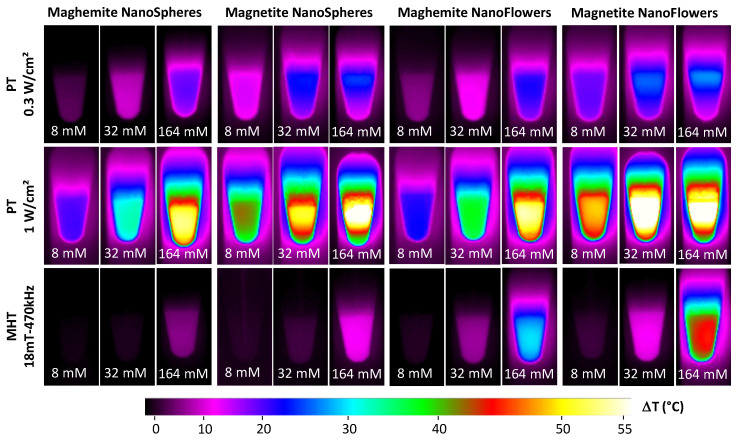
Heating measurements upon photothermal (PT) therapy at 0.3 and 1 W cm^−2^ and magnetic hyperthermia (MHT) at 18 mT and 470 kHz. Representative infrared images of 50 μL of four MNP preparations at Fe concentrations of 8, 32, and 164 mM after 10 min of exposure to the different external stimuli are displayed. Increases in temperature were analyzed for a 1064 nm laser at the power of 0.3 W cm^−2^ (**top row**), 1 W cm^−2^ (**middle row**), and alternating magnetic fields at 18 mT and 470 kHz (**bottom row**).

To investigate the heating potential of all four nanoparticles, maghemite and magnetite nanospheres and maghemite and magnetite nanoflowers were dispersed in water at various concentrations, ranging from [Fe] = 0.5 mM to [Fe] = 164 mM, with increasing steps of 4-folds. The zeta potential in water of citrated nanospheres (magnetite or maghemite) was around −35 mV, while for the citrated nanoflowers (magnetite and maghemite), it was −45 mV, in both cases showing the high colloidal stability of the NPs. For each of four nanoparticles, a solution of 100 µL placed in 1.5 mL tubes was exposed to a 1064-nm laser (with power density set at 0.3 or 1 W cm^−2^) or to 18 mT and a 470 kHz alternating magnetic field. Heating measurements for solutions at iron concentrations of 8, 32, and 164 mM were recorded using an infrared thermal camera, and typical images are shown in Figure 2, which reveal that the heating is increased together with the concentration, yet less marked for photothermal (PT) modality. As expected, heating is increased for PT when exposing at 1 W cm^−2^ compared to 0.3 W cm^−2^, and overall, PT at 1W cm^−2^ is the most efficient setting for all nanoparticles. As previously anticipated, MHT has a generally lower degree of heating performance in respect to PT, even at 0.3 W cm^−2^ at the iron concentration of 8 and 32 mM. Only at 128 mM, MHT becomes worthy of comparison and even higher than PT at 0.3 W cm^−2^, in the case of nanoflowers. Finally, whatever the modality, magnetite nanoparticles appear more efficient than their maghemite counterparts.

**Figure 3:**
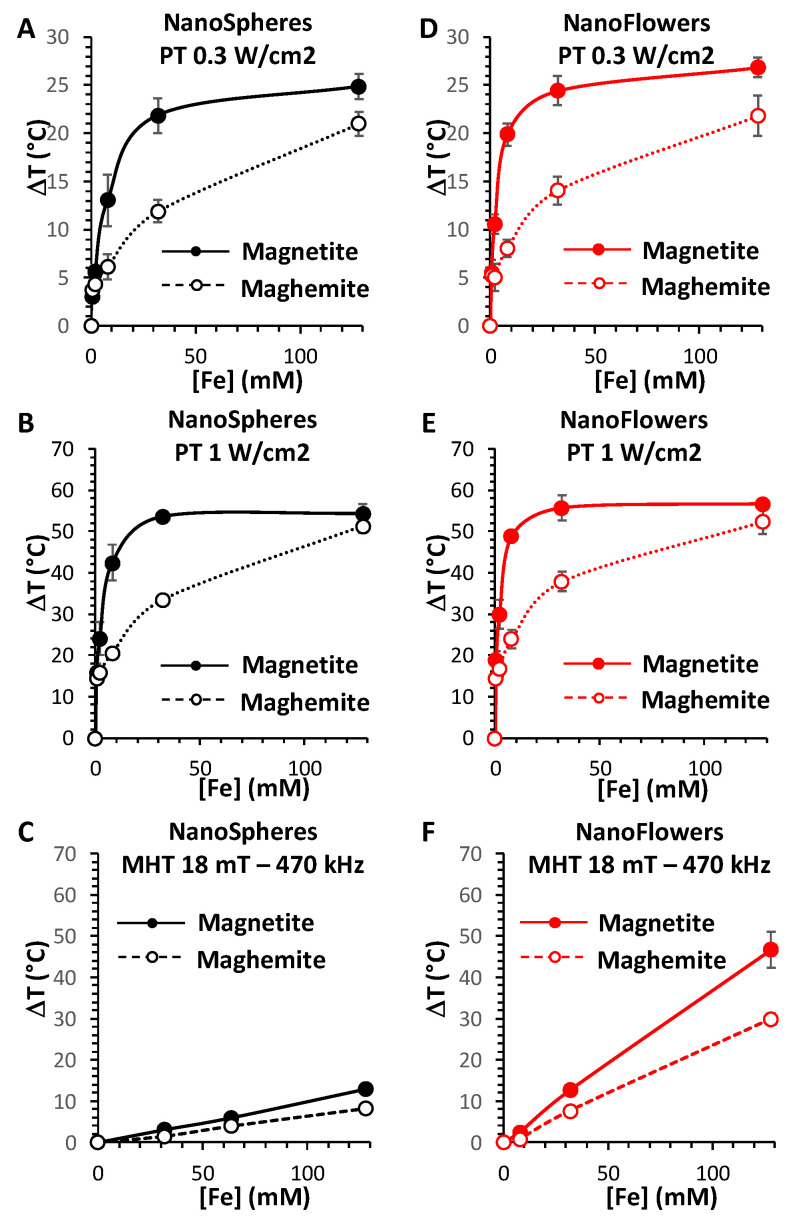
MNP heating performances in water for the PT and MHT heating modalities. Temperature elevation profiles for nanospheres (**A**–**C**) and nanoflowers (**D**–**F**) irradiated with a 1064 nm laser at a power density of 0.3 W cm^−2^ (**A**,**D**), 1 W cm^−2^ (**B**,**E**), and exposed to alternating magnetic fields at 18 mT and 470 kHz (**C**,**F**), according to their concentration, ranging from 0.5 to 168 mM of Fe. All the MNPs are studied in their magnetite (full dotted lines) and maghemite (empty dotted lines) forms.

Furthermore, Figure 3 shows the mean heating profiles of all MNPs in analysis as a function of their concentrations, and for the three heating modalities (PT 0.3 and 1 W/cm^2^, and MHT 18 mT and 470 kHz). Concerning the PT analysis, a saturation effect is produced, starting from concentrations over 20 mM of Fe. By contrast, in the case of MHT, the heating increases linearly with the concentration. This allows the calculation of the specific loss power (SLP), which is independent of the concentration, as defined in the Methods section. SLP is generally used for classifying the heating power of magnetic nanoparticles, with efficient nanoparticles providing SLP over 100 W g^−1^ (in grams of Fe). Another parameter to take into account for this application is the magnetic doses, which are clinically accepted, which is expressed as the product between the used field and frequency (limit set at H × f below 5 × 10^9^ Am^−1^ s^−1^, H being the magnetic field and f the frequency) [31]. The product H × f reached here (18 mT and 470 kHz) equals 6.7 × 10^9^ Am^−1^ s^−1^, which is close to the clinical limit. In this configuration, the SLP of the maghemite and magnetite nanospheres were calculated to be 58 ± 4 W g^−1^ and 88 ± 4 W g^−1^, respectively. For the nanoflowers, the SLP is significantly increased, to 215 ± 10 W g^−1^ and 373 ± 21 W g^−1^, for maghemite and magnetite, respectively.

The highest temperature increase (T) measured was in the range of 50 to 60 °C, reached only for magnetite nanoflowers for MHT at the highest concentration (128 mM of Fe), and reached for all the nanoparticles for PT at 1 W cm^−2^ at this concentration. The striking difference in PT corresponds to the concentration at which this heating is reached: 32 mM of Fe for the magnetite nanospheres and even lower, at almost 8 mM, for the magnetite nanoflowers; in contrast, this ΔT was achieved only at 128 mM for both maghemite nanospheres and nanoflowers.

For PT, the clinical limit of the laser dose is not clearly determined yet and it is possible to find quite a variability reported in literature. Many preclinical studies work with a laser at 1 W cm^−2^, and sometimes even up to 3 or 5 W cm^−2^. However, to assure totally safe conditions for the surrounding healthy tissues, the lower power density of 0.3 W cm^−2^ is generally admitted [41]. With this setting, the maximum temperature increase that can be reached with PT is around ΔT = 25 °C, and is attained at lower concentrations for the magnetite nanoparticles compared to the maghemite ones, with a concentration-dependent heating profile very similar to the one obtained at 1 W cm^−2^.

Overall, magnetite nanoparticles appear to be much more efficient as PT agents, especially at lower iron doses, far from the saturation. Indeed, concentrations over 10 mM of Fe are not likely to be reached in vivo. For instance, if we compare the ΔT of nanospheres of maghemite to nanospheres of magnetite at 8 mM of Fe, it increases from 6 °C to 13 °C at 0.3 W cm^−2^ and from 20 °C to 42 °C at 1 W cm^−2^; while in the case of nanoflowers of maghemite to nanoflowers of magnetite, the ΔT raises from 8 °C to 20 °C at 0.3 W cm^−2^ and from 24 °C to 49 °C at 1 W cm^−2^.

**Figure 4:**
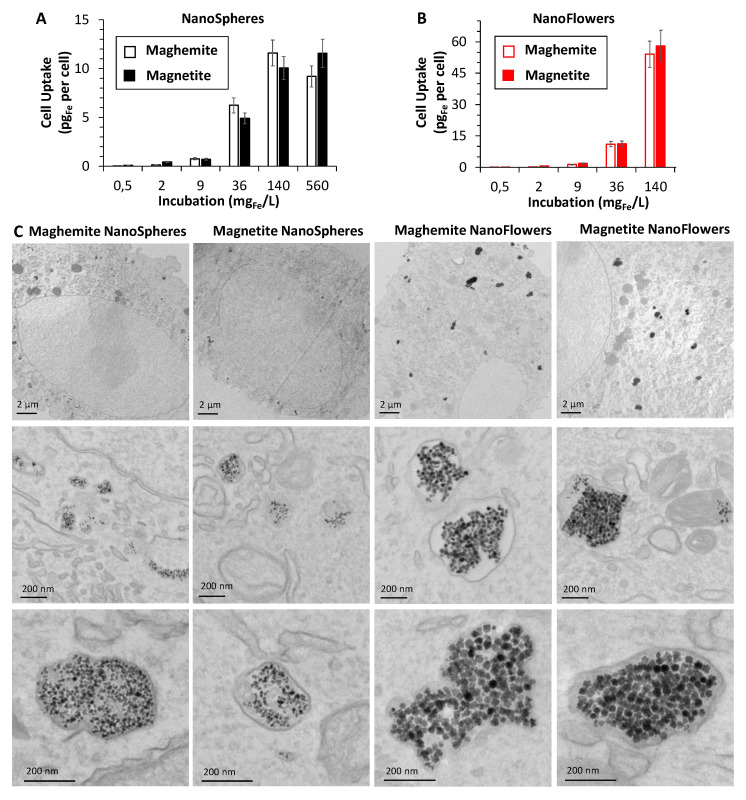
Study of MNPs internalization in vitro inside tumor cells. Chart of the intracellular Fe amounts for nanospheres (**A**) and nanoflowers (**B**) incubated with PC3 prostate cancer cells in their maghemite (empty columns) and magnetite (full columns) forms, at different doses of given extracellular Fe concentrations from 0.5 to 560 mg Fe/L. TEM images showing the intracellular MNP deposits for the four different preparations (**C**) at lower (upper row) and higher magnification (medium row), incubated 30 min at 36 mg Fe/L; in particular, all the nanoparticles are found exclusively within the endosomal compartments (bottom row).

All four nanoparticles were then administered to cancer cells (PC-3 prostatic tumor cells) at increasing doses in the cell culture medium ranging from 0.5 to 560 mg Fe/L, corresponding to concentration from [Fe] = 1 µM to [Fe] = 10 mM. Figure 4A,B presents the cell uptake as a function of the dose, expressed in pg of internalized iron per cell. Nanospheres were less internalized than nanoflowers, probably due to smaller size, reaching the saturation at about 10 pg per cell, while for nanoflowers, a higher amount of 60 pg per cell was attained at the 140 mg/L dose. TEM analysis was performed on samples corresponding to cells incubated at 36 mg Fe/L, revealing a systematic endosomal localization of all nanoparticles (Figure 4C), and a high degree of confinement of the nanoparticles within the endosomes. It also clearly shows that the nanoflowers are more internalized than the nanospheres. Similar internalization of the same nanospheres and nanoflowers, for both with the maghemite crystal phase, was achieved with other cancer cell lines, such as SKOV-3 human ovarian cancer cells [42] or human breast cancer MCF-7 cells [29], respectively.

**Figure 5:**
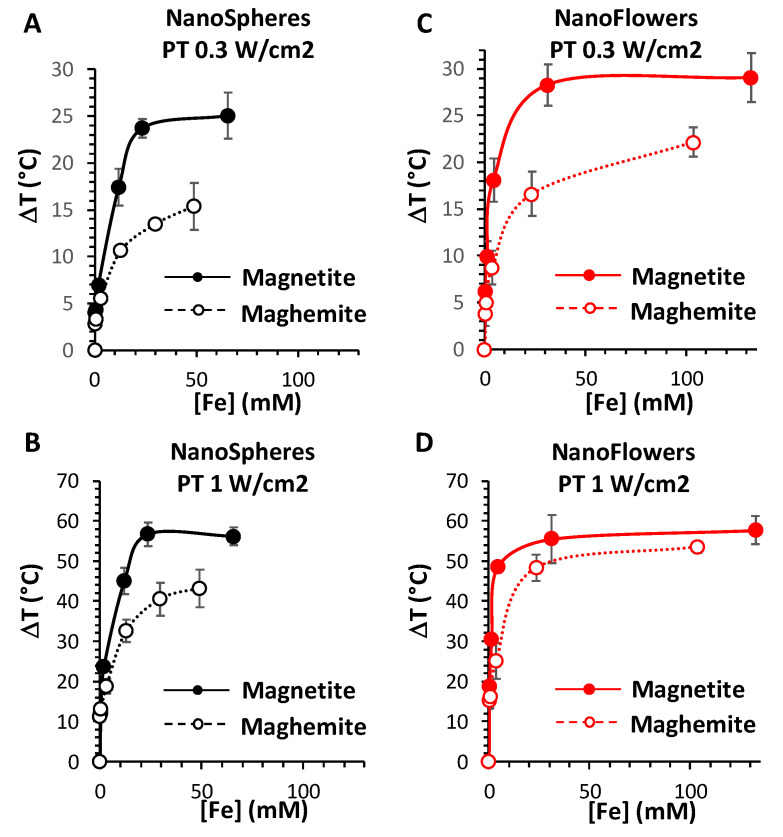
Analysis of the photothermal performances of the MNPs internalized within tumor cells. Temperature elevation profiles for PC3 cells treated with nanospheres (**A**,**B**) and nanoflowers (**C**,**D**) in their magnetite (full dotted lines) and maghemite (empty dotted lines) forms, irradiated with a 1064 nm laser at a power density of 0.3 W cm^−2^ (**A**,**C**) and 1 W cm^−2^ (**B**,**D**), as a function of the iron concentration in the cell suspensions measured afterwards by ICP analysis.

Following nanoparticle internalization, the cells were detached and resuspended in medium in the same configurations used for the heating evaluation of the nanoparticles in solution and without fixation. About 4 million cells were dispersed in 50 µL and the exact Fe concentration in the cells was evaluated subsequently by elemental analysis. In Figure 5, the temperature increase of the cell samples as a function of the cell suspension concentration has been plotted, in a similar way as presented in Figure 3 for the water-dispersed samples. Note that the concentration corresponds to the iron concentration in the tube containing the nanoparticle-loaded cells, and not to the incubation dose. It is noteworthy that each final concentration reached corresponds initially to a given dose of extracellular nanoparticles provided to the cells. Remarkably, the PT heating profiles are very much alike for both situations, evidencing that neither the internalization of the nanoparticles, nor their extensive endosomal confinement, impact their photothermal conversion efficiency, either adversely or beneficially. As a result, the magnetite nanoparticles are again arising as the best PT nanoheaters, this time in the cellular environment, and, between the two shapes of magnetite, the nanoflowers perform even better than the nanospheres. Importantly, no heating could be recorded in MHT modality for any of the cell samples, even for the ones corresponding to the highest concentration. This is due to the high density of nanoparticles within the endosomes, which totally impair their magnetic relaxation, and in turn dramatically decrease their heating power (SLP) [43]. A SLP decrease of over 3-fold and over 10-fold was previously reported for nanospheres and nanoflowers, respectively [31], making it impossible to record a temperature increase, even at concentrations of 60 mM of Fe (for nanospheres) or 120 mM of Fe (for nanoflowers).

**Figure 6:**
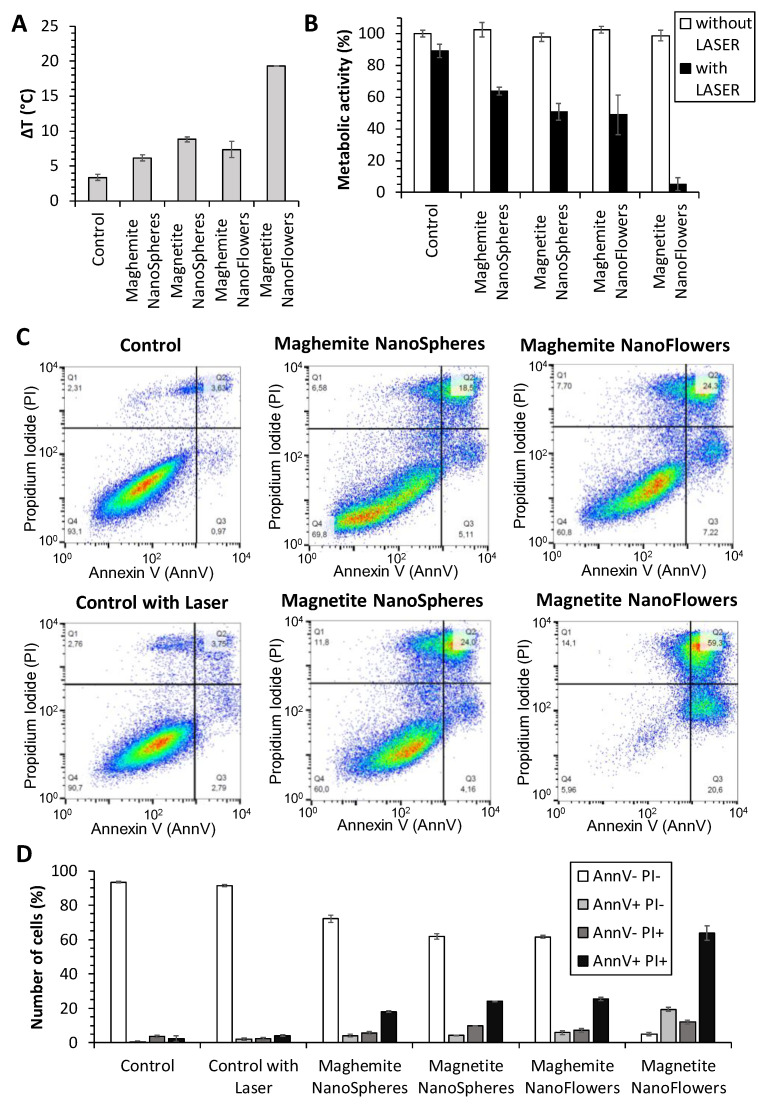
Characterization of the photothermal therapy carried out on PC3 prostatic cancer cells doped with the 4 types of MNPs. Temperature increase achieved in the cell suspensions treated with a 1064 nm laser at a power density of 0.3 W cm^−2^ for 10 min (**A**), and the corresponding outcomes, 24 h after the treatment, analyzed by a metabolic/cytotoxicity test (**B**) with and without the laser irradiation. Annexin V/propidium iodide apoptosis assay (**C**) in the same conditions and the corresponding percentage fractions of each group (**D**) plotted according to the positivity of the staining.

In order to further investigate the MNPs photothermal heating in terms of their therapeutic efficiency, the cells were next incubated with the nanoparticles at one identical dose of 36 mg Fe L^−1^, and then cells were detached and immediately treated with the lower laser dose of 0.3 W cm^−2^ for 10 min, in order to adopt the safer condition for clinical practice. The resulting temperature increases, averaged over three independent experiments, presented in Figure 6A, indicate that only magnetite nanoflowers reach a ΔT over 15 °C, which agrees with the data shown in Figure 5. As a result of the treatment, the metabolic activity of the cells measured 24 h after the treatment is presented in Figure 6B. The test is a measure of the cell mitochondrial redox potential and mirrors directly the cells’ viability. The results show that only magnetite nanoflowers led to a massive cell death, with less than 5% of cells still viable. Here, it is important to notice that the nanoparticles themselves do not trigger any toxicity, leaving the cell viability in absence of the laser being identical to the control without nanoparticles. Moreover, magnetite nanospheres and maghemite nanoflowers exhibit an equivalent effect on cell viability, while maghemite nanospheres are the less efficient ones.

Finally, to further investigate the mechanism of action of the MNPs photothermal therapy, treated cells were also analyzed in relation to their apoptosis and necrosis using annexin V (AnnV) and propidium iodide (PI) staining quantified by flow cytometry. Typical cytometry plots are shown in Figure 6B, unambiguously confirming the massive cancer cell death reached upon treatment with the magnetite nanoflowers (bottom right panel), in which case more than 60% of cells resulting are necrotic and 20% of cells are apoptotic. Figure 6C shows the proportion of cells negative to both annexin V and propidium iodide (AnnV− PI−), representing the viable cells, the proportion of early apoptotic cells (AnnV+ PI−), the one of late apoptotic cells (AnnV+ PI+), and the fraction of necrotic cells (AnnV− PI+). In each nanoparticle-treated condition, it appears that most of the non-viable cells are necrotic. In addition, the relative number of necrotic cells is slightly higher for the magnetite nanoflowers, having also the degree of viable cells reduced to almost zero. These results nicely reflect the impressive efficacy reached with the magnetite nanoparticles treatment.

## 4. Discussion

As the use of iron oxide magnetic nanoparticles in the field of thermal nanotherapies (MHT and PT) has been expanding over years, understanding and quantifying the relation between the characteristics of the nanoparticles, such as their crystal phase (magnetite/maghemite) and size on MHT and PT, as well as providing a comparative study between both modalities for each NP, as described in this paper, has remained necessary.

Impressively, our results show clearly that the crystal phase of the magnetic iron oxide, i.e., magnetite vs. maghemite, has a strong effect on the produced heat by PT. Two times fold in heating is generated by PT at 0.3 and 1 W/cm^2^ with magnetite nanostructures compared to their relative maghemite ones for a same concentration of 8 mM in Fe. For the nanospheres, upon application of a laser of 0.3 W/cm^2^, the temperature elevation ΔT reached 13 °C for the magnetite phase and decreased to 6 °C upon oxidation to the maghemite phase, while for a power of 1 W/cm^2^, ΔT attained 42 °C for the magnetite phase compared to 20 °C for the oxidized maghemite. The same tendency in heating was observed for the magnetic nanoflowers, with a ΔT reaching about 20 °C for the magnetite phase compared to 8 °C for the maghemite at 0.3 W/cm^2^, and attaining 49 °C for the magnetite phase compared to 24 °C for the maghemite at 1 W/cm^2^. The difference in heating obtained by PT before (magnetite) and following oxidation (maghemite) can be clearly attributed to the presence of the absorption band assigned to the intervalence charge transfer localized in the NIR-II biological window of the magnetite phase, which disappears after oxidation. While heating using this band is still limited in the literature, a clear comparative study showing the effect of oxidation has never been reported to the best of our knowledge. Moreover, compared to the available reports on the use of the IVCT of magnetite for PT, the magnetic nanoflowers can be classified as the best available nanoheaters in this field, having a size below 50 nm that is perfectly suitable for biomedical in vivo applications. A high temperature elevation of 33 °C nm was observed in PT using magnetite NPs at a concentration of 6.6 mM in Fe, however, the nanoparticles were 200 nm aggregates formed by the assembly of 21 nm nanoparticles [26]. On the other hand, an elevation of 10 °C was observed using 13 nm iron oxide magnetic NPs at a dose of 10 mg/mL but using a laser power of 14 W/cm^2^ largely exceeding the limits of safety [27,41].

The size and the crystallinity of the nanoparticles have also had a direct impact on the final heating, as the magnetic multicore nanoflowers displayed a superior heating compared to the nanospheres, before and after their oxidation to maghemite (at 1 W/cm^2^, ΔT reached 42 °C for the magnetite nanospheres versus 49 °C for the magnetite nanoflowers and 20 °C for the maghemite nanospheres compared to 24 °C for the maghemite nanoflowers). On the other hand, our results confirm the higher efficiency of magnetic nanoflowers (independently from the crystal phase) compared to the nanospheres in MHT, as already reported [29,30]. For Fe concentration of 128 mM, magnetite nanospheres showed a ΔT of 13 °C compared to 47 °C for the magnetite nanoflowers, which decreased to 8 °C for the maghemite nanospheres and 30 °C for the maghemite nanoflowers. In all conditions, the magnetite phase is also more effective than the maghemite phase for MHT (ΔT of 47 °C for magnetite nanoflowers compared to 30 °C after oxidation to maghemite. In conclusion, magnetite iron oxides appear more efficient in PT compared to MHT and magnetic nanoflowers are better heating agents than nanospheres.

PT has several advantages over MHT, especially for low/medium NPs doses, where PT yields a much more efficient heating than MHT. However, the main disadvantage of PT is that high concentrations lead to saturation. If high heating is required, then MHT must thus be used. This will require high doses of magnetic nanoparticles, and such doses can only match intratumoral injections. Moreover, despite being an efficient thermal modality in solution, in the cell environment, MHT using nanoflowers is totally destroyed, therefore using PT will be the only choice. Impressively and in contrary to MHT, PT maintained the same efficiency in cells as in aqueous solutions. At this stage of understanding, it is obvious that magnetite nanoflowers are the best candidates for PT. Added to that is an even more decisive advantage: magnetite nanoflowers are internalized 5 to 6 times more in cancer cells than their single-core counterpart. On the level of the therapeutic efficacy and at a same dose of iron administered to cancer cells, these results translated absolutely in favor of magnetite nanoflowers, with the maghemite nanoflowers being as effective as the magnetite nanospheres. Only the magnetite nanoflowers led to complete cancer cell eradication, while maghemite nanoparticles obtained by co-precipitation, which are among the most used in biomedical (ease and aqueous green synthesis), are not suitable for therapy in these settings.

## 5. Conclusions

To summarize, in this paper we investigated the thermal modalities in MHT and PT in aqueous solution and in cancer cells of two renown magnetic nanoparticles in the biomedical field. Twelve nm magnetic nanospheres and 27 nm magnetic multicore nanoflowers formed by the assembly of 11 nm cores, one among the best nanoheaters in MHT, were synthesized and characterized in the magnetite and the maghemite crystal structures. Our results in solution showed that the magnetite structure independently of the shape are the best heaters in MHT and PT. While in cells, MHT was inhibited for all NPs, and PT appeared very efficient at low doses and remarkably preserved. Moreover, magnetite nanoflowers showed a remarkable increase in cell uptake compared to the nanospheres and a spectacular total cell death during antitumoral therapy at a reasonable laser power density (0.3 W/cm^2^). These results open up new perspectives on the use of magnetic nanoparticles as multimodal platforms for theranostic applications, and more particularly in the field of thermal nanotherapies. In this proof of concept work, we compared two shapes of nanoparticles of similar chemical composition that differed by their crystallinity since they were produced using two different synthesis methods. They also differ by their size, which can also impact the optical properties of the nanostructures and their PT. In order to rationalize and get more understanding, in the near future, we would like to investigate on PT different parameters, including the fine structure of the magnetic core (monocore vs. multicore), the size, and the chemical composition of nanoparticles produced using the same synthesis protocol. Although the oxidation of magnetite was very slow at 4 °C, it should be avoided in order to extend its use to other biomedical applications. Engineering the surface of the NPs using different ligands and coatings would be a methodology.
